# The *fasn-1(g14ts)* allele is a Gly1830Arg missense mutation in *C. elegans* FASN-1

**DOI:** 10.17912/micropub.biology.000244

**Published:** 2020-05-05

**Authors:** Xiaofei Bai, David Woodbury, Andy Golden

**Affiliations:** 1 Laboratory of Biochemistry and Genetics/NIDDK/NIH

**Figure 1. Two temperature-sensitive alleles in the C. elegansfasn-1 gene disrupt eggshell formation and cause embryonic lethality:**
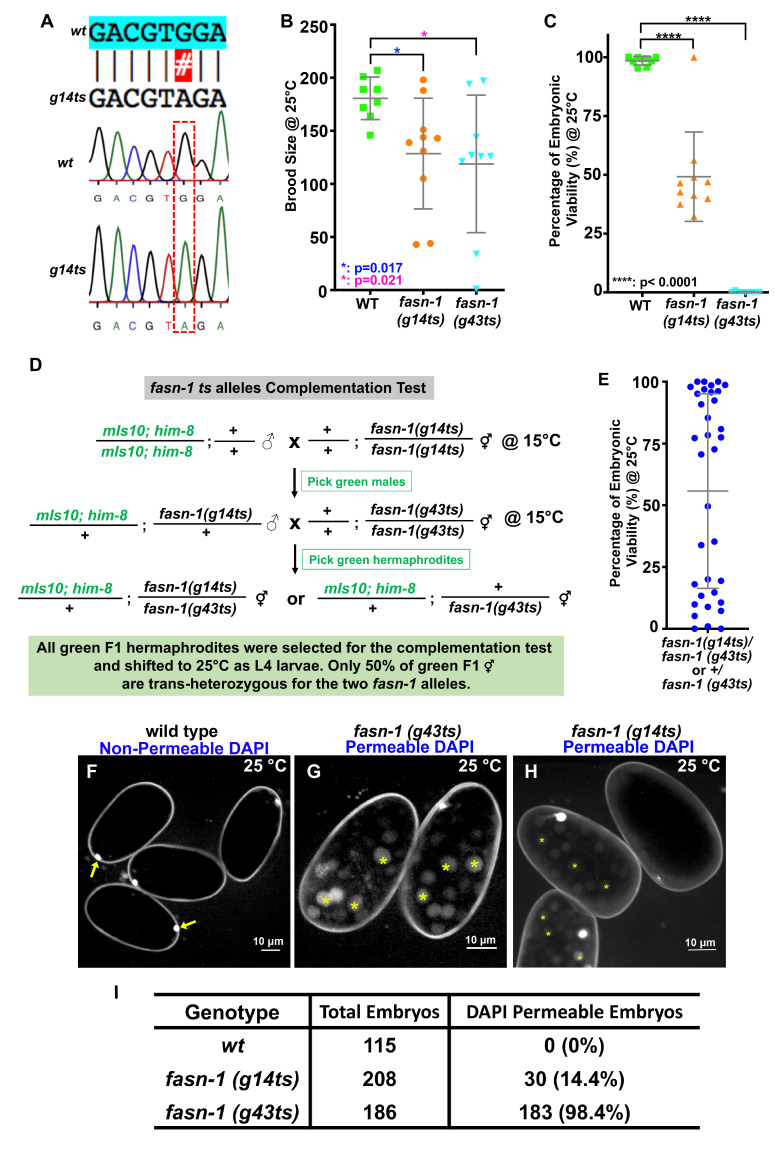
(A) A missense mutation (GGA to AGA, Gly1830Arg) was identified by Sanger sequencing of the *fasn-1* gene from the *g14ts* mutant. (B) Brood size was reduced in both *fasn-1* temperature sensitive alleles at non-permissive temperature (25°C) compared with wild type. (C) The percentage of embryonic viability was reduced in the *fasn-1* ts alleles. (D) The workflow of the complementation test for *fasn-1* ts alleles. The F1 hermaphrodites were then shifted to 25°C as L4 larvae to test for complementation. (E) The percentage of embryonic viability in the progeny of the green F1 hermaphrodites shown in panel D. (F-H) Representative images of DAPI stained embryos of wild type and the two *fasn-1* temperature sensitive mutants. DAPI staining of zygotic chromatin (yellow asterisks, G, H) was apparent in *fasn-1* temperature sensitive embryos, while only the first polar body in wildtype embryos is DAPI-stained (yellow arrows, F). (I) Quantification of DAPI staining of zygotic chromatin in the different strains. P-values: * = 0.017 (B); * = 0.021 (B); **** <0.0001 (C) (t-test). Scale bars are indicated in panels F-H.

## Description

We had previously identified the causative mutation in *emb-14(g43ts)* as an Ala1425Thr missense mutation in the *C. elegans fasn-1* gene on LGI (Jaramillo-Lambert *et al.* 2015). At that time, it was suggested that the *emb-14* gene be renamed *fasn-1*. FASN-1 is a fatty acid synthase orthologous to human FASN (Lee *et al.* 2010). The *g43ts* allele is a tight, highly penetrant temperature-sensitive allele. There are presumably two other alleles, *g14ts* and *fr8*. We characterized the osmotic sensitivity of *g14ts* embryos, as well as the brood size and embryonic viability of the *g14ts* allele. We also demonstrated that *g14ts* failed to complement the tight temperature-sensitive allele, *g43ts*. We also determined the sequence alteration of *g14ts* to be a Gly1830Arg missense mutation upon sequencing PCR fragments that covered the entire *fasn-1* gene (Fig. 1A). *fr8* was previously identified as an M241I missense mutation in the *fasn-1* gene. Interestingly, 40% of *fasn-1(fr8)* embryos fail to hatch (Lee *et al.* 2010).

The brood size of the *g14ts* and *g43ts* strains were variable but statistically different than N2, the wildtype strain (Fig. 1B). Brood size was determined by moving mothers each day to fresh plates and counting the number of hatched larvae and unhatched dead embryos on each plate 24 hrs after moving the mothers. Daily transfers were performed until the mothers stopped laying fertilized embryos. Brood size was defined as the sum of hatched larvae and unhatched dead embryos. Embryonic viability (% hatching) at 25°C was quite variable for *g14ts* (Fig 1C). However, *g43ts* embryos failed to hatch at 25°C, while the wildtype embryos hatched >98% (Fig. 1C).

To confirm that *g14ts* was an allele of *fasn-1*, we did a complementation assay with *g43ts* (Fig. 1D). We used males marked with a pharyngeal GFP marker (*mIs10*) to confirm that cross progeny were being analyzed. Half of the F1 hermaphrodites should be *g14ts/g43ts* while half should be just *g43ts/+*. All F1 animals were then shifted to 25°C as L4 larvae, and the embryonic viability of each mother was quantified (Fig. 1E). Embryonic viability was bimodal, likely reflecting that half the F1 failed to complement at 25°C, and the other half were *g43ts/+* mothers with high levels of embryonic hatching.

To quantify the defective eggshells of the *fasn-1* mutants, N2, *g14ts,* and *g43ts* were subjected to DAPI staining. Young gravid hermaphrodites were washed in M9 buffer three times and dissected with 23 G x 3/4” needles. Different stage mitotic embryos were transferred to a homemade hanging drop chamber made by molten Vaseline (Edgar and Goldstein 2010). The chamber was filled with Blastomere Culture Medium (BCM) with 5 μg/ml DAPI. The images were captured by high-resolution confocal microscopy with a Nikon 60 X 1.2 NA water objective with 1 μm z-step size. Images were generated by custom Fiji Image J>Stacks>Z project (Linkert *et al.* 2010, Schindelin *et al.* 2012). As expected, N2 embryos were not permeable to DAPI (other than the first polar body; Fig. 1F) while *fasn-1(g43ts)* embryos were highly permeable. *fasn-1(g14ts)* embryos were variably osmotically sensitive with only 14.4% showing DAPI permeability (Fig. 1G, H). *fr8* was also reported to be permeable to the lipophilic dye Nile red (Lee *et al.* 2010). Of the three existing alleles, *g43ts* is the tightest and most penetrant. Suppressor screens with this allele would be quite straight forward.

## Reagents

N2: Bristol (wild type)

GG14: *fasn-1(g14ts) I*

GG43: *fasn-1(g43ts) I*

TY4236*:* *him-8(e1489) IV; mIs10 V.*

All strains are available from the CGC.
